# Deconstructing 3D growth rates from transmission microscopy images of facetted crystals as captured *in situ* within supersaturated aqueous solutions

**DOI:** 10.1107/S1600576724008173

**Published:** 2024-09-25

**Authors:** Cai Y. Ma, Chen Jiang, Thomas P. Ilett, Thomas A. Hazlehurst, David C. Hogg, Kevin J. Roberts

**Affiliations:** a Centre for the Digital Design of Drug Products, School of Chemical and Process Engineering University of Leeds LeedsLS2 9JT United Kingdom; b School of Computing University of Leeds LeedsLS2 9JT United Kingdom; Shiv Nadar Institution of Eminence, India

**Keywords:** l-glutamic acid, crystal morphological images, facet-based crystal-growth-rate measurements, polyhedral image analysis, 3D crystal growth evolution, *in situ* characterization, transmission microscopy, supersaturation

## Abstract

Here, a morphologically based approach is used for the *in situ* characterization of 3D growth rates of facetted crystals from the solution phase. Crystal images of single crystals of the β-form of l-glutamic acid are captured *in situ* during their growth at a relative supersaturation of 1.05 using transmission optical microscopy. The crystal growth rates estimated for both the {101} capping and {021} prismatic faces through image processing are consistent with those determined using reflection light mode [Jiang, Ma, Hazlehurst, Ilett, Jackson, Hogg & Roberts (2024 ▸). *Cryst. Growth Des.***24**, 3277–3288]. The growth rate in the {010} face is, for the first time, estimated from the shadow widths of the {021} prismatic faces and found to be typically about half that of the {021} prismatic faces. Analysis of the 3D shape during growth reveals that the initial needle-like crystal morphology develops during the growth process to become more tabular, associated with the Zingg factor evolving from 2.9 to 1.7 (>1). The change in relative solution supersaturation during the growth process is estimated from calculations of the crystal volume, offering an alternative approach to determine this dynamically from visual observations.

## Abstract 2

The crystal growth rate of the basal plane {010} face of β-form l-glutamic acid is estimated from the shadow widths of the prismatic {021} faces in optical microscopy images. This new approach for the *in situ* characterization of 3D crystal shape and size is part of wider research into digital crystallization process engineering, encompassing machine learning, morphological population balance modelling and crystallizer hydro­dynamics simulation.

## 1. Introduction

Understanding, controlling and predicting the size and shape of crystalline materials are of critical importance in the digital design of particulate materials, particularly in relation to their manufacturing and formulation processes (Anuar *et al.*, 2022 ▸). Morphological population balance (MPB) models (Ma *et al.*, 2008 ▸; Ma & Roberts, 2018 ▸, 2019 ▸) can be important tools in the digital design of the crystallization process (Camacho Corzo *et al.*, 2020 ▸) in terms of predicting and controlling the temporal evolution of crystal size and shape. Whilst knowledge of the facet-specific growth rates of all crystal habit faces together with their individual growth interface kinetics is a key input for MPB modelling, the experimental determination of these facet growth rates can pose significant challenges.

On-line imaging systems have previously been used in crystallizers for determining crystal growth kinetics (*e.g.* Hermanto *et al.*, 2008 ▸; Huo & Guan, 2021 ▸; Ma & Wang, 2012 ▸; Ma *et al.*, 2007 ▸; Ochsenbein *et al.*, 2014 ▸, 2015 ▸; Schöll *et al.*, 2007 ▸; Wang *et al.*, 2007 ▸; Wu *et al.*, 2016 ▸), as well as for quantifying the effect of changing operating conditions and solvents on processing behaviour. Such techniques can also monitor variations of crystal size and shape during the processes (*e.g.* Calderon De Anda *et al.*, 2005*a* ▸,*b* ▸,*c* ▸; Gao *et al.*, 2018 ▸; Huo *et al.*, 2016 ▸; Li *et al.*, 2006 ▸, 2008 ▸; Khan *et al.*, 2011 ▸). However, such on-line crystal images are typically of lower resolution and can only provide single projected views of the crystals, which are captured temporally from within a population of crystals. Hence, these techniques currently lack the ability to track the progression and development of individual crystals spatially over time. In contrast, the images captured *in situ* within single-crystal growth systems, using high-resolution optical microscopy, can facilitate the measurement of the facet growth rates along the individual face directions and hence can be much more suitable for 3D growth rate measurement (*e.g.* Nguyen *et al.*, 2014 ▸, 2017 ▸, 2021 ▸; Toroz *et al.*, 2015 ▸; Camacho *et al.*, 2017 ▸; Offiler *et al.*, 2022 ▸; Sacchi *et al.*, 2023 ▸; Kitamura & Ishizu, 1998 ▸, 2000 ▸; Ochsenbein *et al.*, 2015 ▸; Jiang *et al.*, 2024 ▸).

Previously, crystal-growth kinetic studies (Ristic *et al.*, 1993 ▸, 1996 ▸) have examined the growth of selected single-crystal surfaces using microscopic images, with the corresponding crystal morphology being determined through comparison with morphological predictions (*e.g.* Hammond *et al.*, 2006 ▸; Anuar *et al.*, 2022 ▸; Clydesdale *et al.*, 1996 ▸) based upon the material’s crystallographic structure. Such techniques have been mostly *ex situ* and have not always been compatible with the examination and characterization of all the crystal habit faces within the material’s external crystal morphology, hence providing significant challenges to the simultaneous characterization of the growth rates of all the crystal habit faces. Although a number of specialist techniques such as *in situ* interferometry (Li *et al.*, 2023 ▸) have been used to measure the growth rate of *e.g.* the {101} faces of potassium di­hydrogen phosphate, simultaneous measurements of the growth rates of all of the crystal habit faces encompassed within a single crystal have not yet been achieved. Similarly, laser confocal microscopy with differential interference contrast microscopy (Maeki *et al.*, 2020 ▸) has been used for real-time measurements of the growth rate of the {110} face of lysozyme, but again not all habit faces could be measured simultaneously. Confocal microscopy has been used to measure the growth rates of ice crystals *in situ*, but the longitudinal and transverse growth rates measured were not found to be very repeatable, displaying a high variance between four repeated runs. The technique was also found to lack the ability to acquire 3D time-lapse images (Marcellini *et al.*, 2016 ▸). All these limitations have conspired to limit the ability of researchers to acquire the 3D growth rate data that are needed for industrial crystallization process control and scale up.

**Figure 1:**
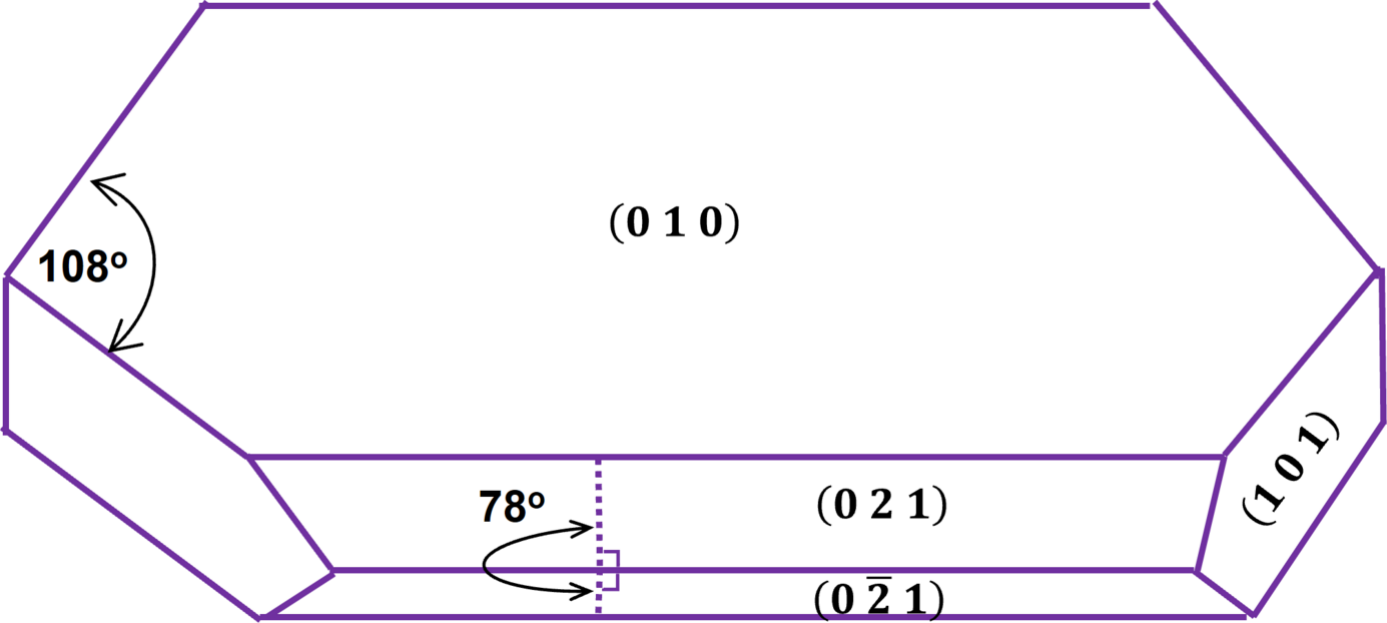
A schematic drawing of β-LGA crystal morphology with some face-to-face angles.

It is well known that l-glutamic acid (LGA) displays a well defined external elongated plate-like crystal morphology (Fig. 1 ▸), and, as a result, this has been widely used as a model compound in crystallization process research (Huo & Guan, 2021 ▸; Gao *et al.*, 2018 ▸; Tahri *et al.*, 2016 ▸; Huo *et al.*, 2016 ▸; Ochsenbein *et al.*, 2014 ▸, 2015 ▸; Ma & Wang, 2012 ▸; Hermanto *et al.*, 2008 ▸; Wang *et al.*, 2007 ▸; Schöll *et al.*, 2007 ▸; Ma *et al.*, 2007 ▸; Li *et al.*, 2006 ▸; Hammond *et al.*, 2005 ▸; Calderon De Anda *et al.*, 2005*c* ▸; Ono *et al.*, 2004 ▸; Liang *et al.*, 2004*a* ▸,*b* ▸; Kitamura & Ishizu, 2000 ▸; Kitamura, 1989 ▸; Khan *et al.*, 2011 ▸). Previous studies on estimating growth-rate measurements of LGA crystallization from solution have usually utilized techniques such as focused beam reflectance measurement (Hermanto *et al.*, 2008 ▸; Lindenberg & Mazzotti, 2009 ▸; Schöll *et al.*, 2007 ▸) to generate chord length distributions to indicate the progression of the crystal growth process. Laser light scattering (Ono *et al.*, 2004 ▸) has been used to produce 1D volume equivalent size data for growth-rate estimation. Pragmatism has necessitated utilizing a spherical crystal shape assumption for the estimation of particle size, even for needle-like crystals such as the β-form of LGA (β-LGA), but this approach has typically only provided a 1D measure of the crystal growth process. Recent advances in the development of in-process imaging systems including particle vision and measurement (Mettler Toledo, 2020 ▸), and the Perdix and BlazeMetrics (2021 ▸) imaging systems (*e.g.* Calderon De Anda *et al.*, 2005*a* ▸,*b* ▸,*c* ▸; Camacho *et al.*, 2017 ▸; Gao *et al.*, 2018 ▸; Huo & Guan, 2021 ▸; Huo *et al.*, 2016 ▸; Kitamura & Ishizu, 1998 ▸; Li *et al.*, 2006 ▸, 2008 ▸; Ma *et al.*, 2007 ▸; Ma & Wang, 2012 ▸; Nguyen *et al.*, 2014 ▸, 2017 ▸, 2021 ▸; Ochsenbein *et al.*, 2014 ▸, 2015 ▸; Sacchi *et al.*, 2023 ▸; Wang *et al.*, 2007 ▸; Turner *et al.*, 2019 ▸), have led to the online capture of images of crystals during crystallization, enabling an estimation of their 2D (length and width) growth rates. However, measurements of the slow rates of growth displayed by the larger surface area of crystal habit faces can be quite challenging. For example, measurements made in the width direction, such as the {021} face direction of β-LGA crystals, were found to have a high variance (Wang *et al.*, 2007 ▸) or could not to be reliably obtained (Kitamura & Ishizu, 1998 ▸). Growth-rate measurements in the vertical direction, such as the {010} face direction of β-LGA crystals, pose significant challenges and, to date, have not been successfully achieved (Jiang *et al.*, 2024 ▸; Kitamura & Ishizu, 1998 ▸).

In a recently published paper (Jiang *et al.*, 2024 ▸), an automated process using a state-of-the-art computer-vision and machine-learning method was used to segment crystal images and, through this, measure *in situ* the crystal growth rates and associated kinetic mechanisms for the capping {101} and prismatic {021} faces of β-LGA crystallized from the solution phase. The accuracies and efficiencies of this new crystal-measurement approach were confirmed through demonstration of equivalent accuracy over a much shorter time compared with existing manual and semi-automatic methods. However, the approach did not produce the crystal growth rates in three dimensions *i.e.* for all the habit faces, most notably for the slow-growing {010} faces. Although there have been attempts to determine the full 3D growth kinetics of LGA crystals for all of the individual face directions growing in a crystallizer using image systems with multiple cameras (Wu *et al.*, 2016 ▸; Zhang *et al.*, 2017 ▸; Rajagopalan *et al.*, 2017 ▸), direct measurements of the facet growth rate for the {010} basal plane face of β-LGA crystals have not to date been available within the published literature. Overall, simultaneous measurements of crystal growth rates of all habit faces are crucial for crystal size and shape control during crystallization processes, hence producing desirable precision crystals for downstream particle processes. The assumption of 1D volume equivalent spherical or 2D rod/needle-shaped crystals will always be limiting due to these models’ inherent loss of 3D size/shape information associated with the facets of real 3D crystals, and with it, the surface properties of the different crystal faces and hence their impact upon particle processability and product performance (Sun, 2009 ▸).

In this study, crystal growth rates in three dimensions have been obtained for the first time through measurements of the capping {101} and prismatic {021} faces, and also the basal {010} faces, of β-LGA crystals simultaneously. The crystal growth was measured *in situ* under transmission light mode [text omitted to avoid apparent repetition]. The 3D growth data were used to estimate the crystal size and shape evolution with time, and the measured growth rates of the capping and prismatic faces have been compared with those previously determined using reflective light mode (Jiang *et al.*, 2024 ▸).

## 2. Experimental methods

### 2.1. Materials

LGA with a purity of ≥99% was purchased from Sigma–Aldrich and directly used without any further purification. Distilled water was produced in house. β-LGA crystallizes in the space group *P*2_1_2_1_2_1_ in a tetramolecular unit cell. The crystallographic data are listed in Table 1 ▸ and the external crystal morphology is shown in Fig. 1 ▸. The latter highlights the interplanar angles between the (101) and (101) faces and between the (021) and (021) faces as being 108 and 78°, respectively.

### 2.2. Experimental apparatus

**Figure 2:**
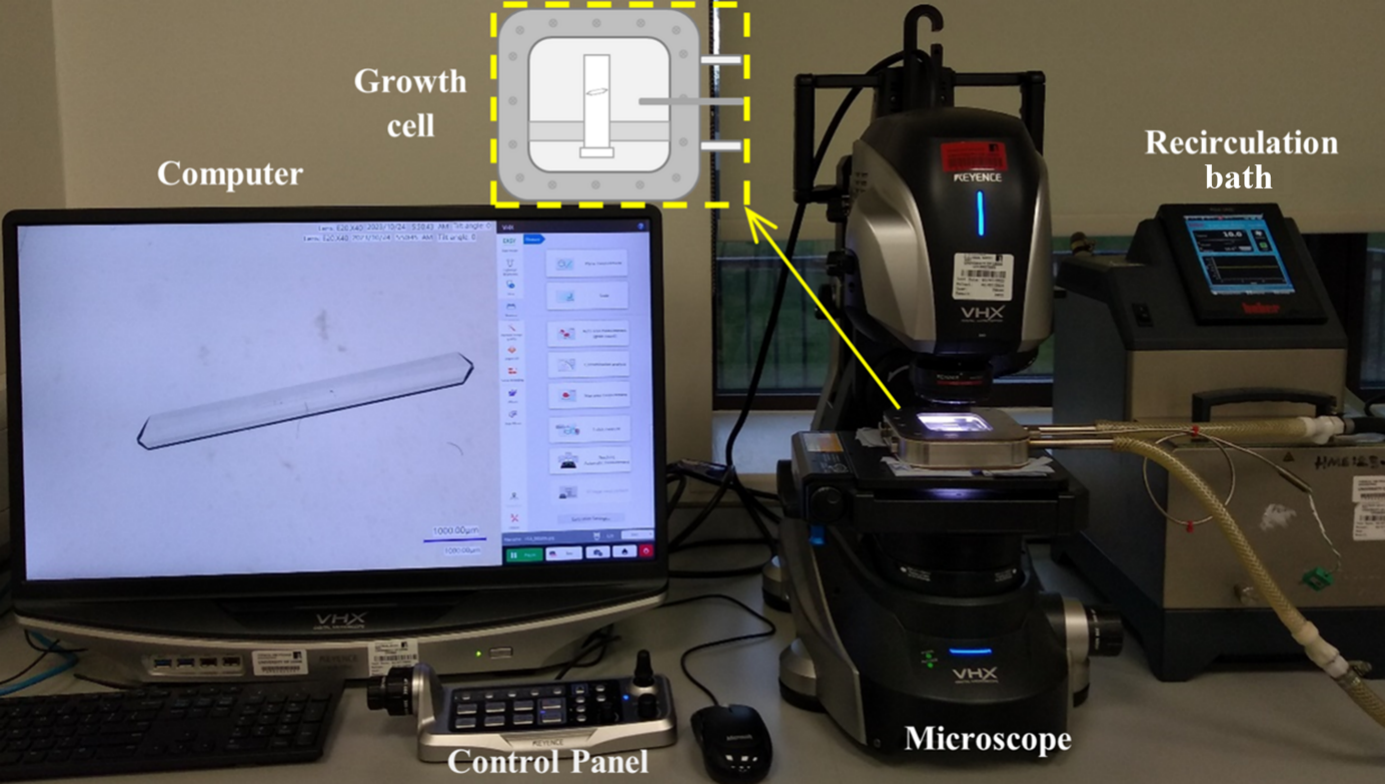
The experimental setup for the growth-rate measurements of β-LGA single crystals in individual face directions. A single-crystal seed was placed in the growth cell and maintained at the target temperature by the recirculation bath. A digital microscope in transmission light mode recorded images at a fixed time interval of 5 min.

A crystal growth cell with temperature control enabled by a recirculating thermostatic bath (Julabo F25) was set up with a Keyence VHX7000 digital microscope to capture high-quality single-crystal images of β-LGA. The setup is summarized in Fig. 2 ▸ and consists of a temperature-controlled glass cuvette cell (Camacho *et al.*, 2017 ▸; Nguyen *et al.*, 2014 ▸), a digital microscope (Keyence, 2021 ▸) integrated with zoom lenses (20×–100×, 100×–500× and 500×–2500×) with a numerical aperture of 0.9, a 1/1.7 inch 4K CMOS image sensor (108 megapixels) camera, and a computer with image capturing and analysis software. The UV cuvette glass cell for crystal growth had a volume of 0.5 ml with corresponding internal sizes of 54 × 10 × 1 mm, and was submerged in a small shallow cell filled with water from the recirculation bath (Fig. 2 ▸) for temperature control. The thickness of the solution in the growth cell was less than 1 mm, with the solution temperature controlled by the circulating water surrounding the cell. The solution itself was stagnant. The Keyence digital microscope has two light modes: reflection and transmission. This study follows the same experimental procedure and parameters as studies using reflection mode (Jiang *et al.*, 2024 ▸), except that it uses transmission light mode. The transmission light mode with zero tilting angle provided a vertical incidence of LED lighting. The effect of possible light beam divergence was found to be limited (see Fig. S1 of the supporting information for further details).

### 2.3. Data acquisition

*In situ* crystal growth of single β-LGA crystals in the individual face directions at a relative solution supersaturation (σ) of 1.05 was measured. The relative supersaturation can be defined as
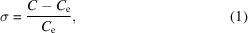
where *C* is the solute concentration and *C*_e_ is its solubility at the same temperature (equilibrium concentration). The solubility of β-LGA in distilled water reported in the literature (Khellaf *et al.*, 2021 ▸) was used for this study. The (010) face of the β-LGA seed crystals was found to lie close to and parallel to the cuvette base surface, consistent with this habit plane having the highest surface area. However, the seed crystal did not sit directly on or stick to the base glass surface of the cell. Instead there was a layer of solution between the (010) face and the base plate, which might explain why the final crystal was found to be quite symmetric with respect to the top (010) and bottom (010) faces (Fig. S2). This was consistent with the findings in the literature (Dold *et al.*, 2006 ▸) with a similar experimental configuration to the current study.

Single crystals of β-LGA were prepared by slow evaporation from a solution containing 10 g of LGA in 1 l of water and used for growth-rate measurement experiments [see Jiang *et al.* (2024 ▸) for further details]. The LGA solution was prepared by dissolving 35 g of the solute LGA in 1 l of de-ionized water (based on the solubility at 67°C) and then transferred into a cuvette cell using a pipette. A seed crystal of β-LGA was placed into the cuvette cell, which was then carefully and rapidly sealed and firmly fixed at the bottom part of the growth cell. The water bath was set at a temperature of 67°C to limit the potential for any secondary nucleation. After that, the solutions were cooled to 46°C to generate a σ of 1.05 and kept at that temperature until the end of the growth process. Images were captured using automatic focus at a constant time interval of 5 min.

### 2.4. Data analysis

#### 2.4.1. Image analysis of facet growth

**Figure 3:**
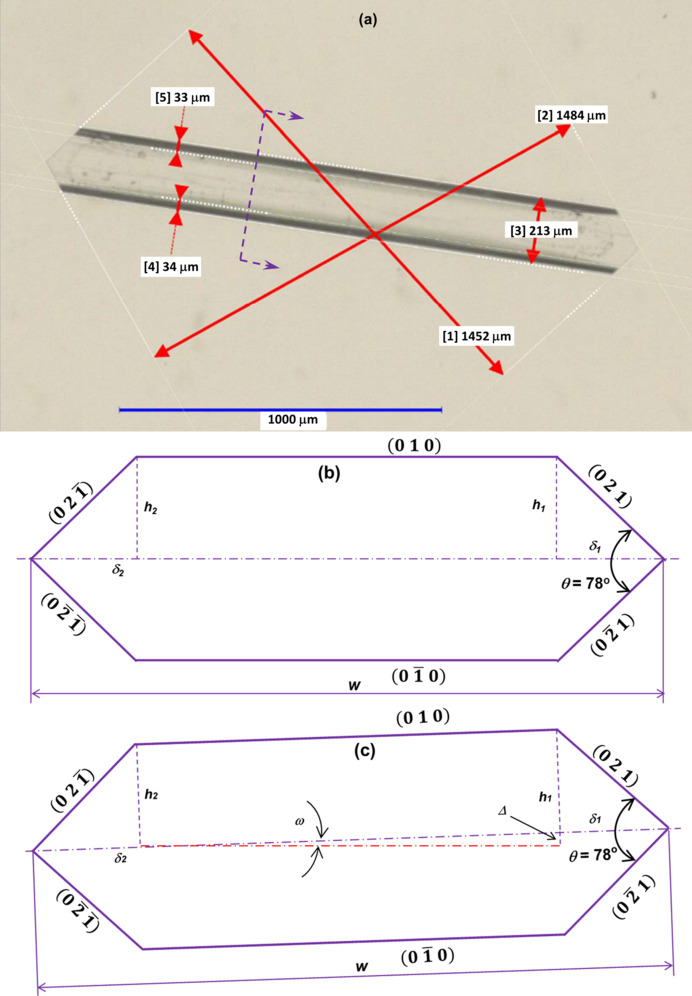
(*a*) A typical image under transmission light mode with the distances of faces (101)/(101) [1], (101)/(101) [2] and (021)/(021) [3], and the two measured distances of faces (021) and (021) between the edges of faces (010)–(021) and (021)–(021) [4] and faces (021) and (021) between the edges of faces (010)–(021) and (021)–(021) [5]. (*b*) A schematic drawing of the section cutting across {021} faces [dashed purple line with arrows in (*a*)] to show the parameters for estimating the distance between {010} faces. (*c*) A schematic drawing to estimate the tilt angle (ω) of {010} faces if *h*_1_ ≠ *h*_2_. The values in (*a*) were converted from the measured pixels on the basis of the scale bar after calibration.

A manual image-analysis method was used to draw parallel lines along the edges of two paired crystal faces in a crystal image, hence determining the normal distance within the pixelated image between these two lines (Jiang *et al.*, 2024 ▸). The actual distance in length units was found by considering the calibrated actual pixel size. The procedure was repeated for the other paired faces and all crystal images recorded with σ = 1.05. In this study, Keyence measurement software (Keyence, 2021 ▸) was used to obtain the actual distances between paired faces [(101) and (101), (101) and (101), and (021) and (021)] and also the thicknesses of the shadow areas associated with the projection of the inclined {021} habit surfaces, as shown in Fig. 3 ▸(*a*).

The β-LGA growth normal to the basal plane {010} faces, *i.e.* in the vertical direction with respect to the optical axis of the microscope, was determined from the images captured under transmission mode by the shadow widths, δ_1_ and δ_2_, of the {021} faces with the angle θ, as shown in Fig. 3 ▸:
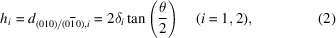
where *d*_(010)/(0
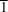
0),*i*_ and *h_i_* are estimated heights in the {010} face normal direction.

The two distances were averaged and then used for growth-rate determination of {010} faces. A similar technique was used to estimate the height, and hence the height growth rate, of tolfenamic acid crystals by Sacchi *et al.* (2023 ▸).

As mentioned above, the final crystal was quite symmetric with respect to the top (010) and bottom (010) faces (Fig. S2). This would be consistent with the existence of a thin solution layer between the (010) crystal face and the base surface of the cuvette, which allows the (010) face to grow. If the {010} faces and the base surface were not completely parallel to each other [Fig. 3 ▸(*c*)], the values δ_1_ and δ_2_ would produce two different heights (*h*_1_ and *h*_2_) if δ_1_ ≠ δ_2_. With a difference of *h*_1_ − *h*_2_, as shown in Fig. 3 ▸(*c*), the tilt angle (ω) of the {010} faces with respect to the horizontal flat surface of the cuvette was estimated as
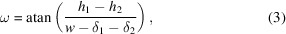
where *w* is the distance in the width direction.

The estimated tilt angles were used to provide an indication as to whether the {010} faces were lying completely parallel with respect to the base surface of the cuvette (*i.e.* in such a case, the tilt angle would be 0°). The tilt-angle estimation was based upon the assumption that the symmetrical faces of prismatic {021} or basal plane {010} faces were growing with the same rates.

#### 2.4.2. Determination of LGA’s 3D crystal growth rates and morphological evolution

As the single-crystal seed grows within a growth cell, the solute concentration decreases due to the consumption of the solute through the growth process. Therefore, only the early initial stage of the growth process can be expected to give a good linear fit between the measured paired distances associated with crystal facet growth as a function of time. This linear fit represents the growth rates at the prescribed supersaturation (Jiang *et al.*, 2024 ▸), *i.e.* consistent with the situation that envisages only a slight consumption of the solute during this early stage. The estimated growth rate was defined as half of the growth rate of the paired faces. Typically, at the later stages, it was found that the growth rates would deviate from a linear relationship, reflecting solute depletion.

With the measured centre-to-normal distances of the three facet forms, *i.e.* {101}, {021} and {010}, the resultant 3D crystal sizes and shapes were plotted using *VisualHabit* in *Mercury* (Macrae *et al.*, 2020 ▸). The 3D shape quantifying Zingg factor (Liu *et al.*, 2008 ▸) Fz, defined as
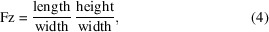
was calculated as a measure of the overall particle morphology (Liu *et al.*, 2008 ▸), *i.e.* needle-like crystals when Fz > 1 or plate-like crystals when Fz < 1.

#### 2.4.3. Estimation of solution supersaturation from crystal volume changes with time

The volume of β-LGA crystals and their evolution over time were calculated by dividing the polyhedral crystal into *N* adjacent pyramids (*N* = number of crystal faces). The base sides of these pyramids were the individual (*hkl*) faces with the facet surface area *S*_*hkl*_ [surface area of face (*hkl*)], and the radial distances were the crystal centre-to-face-normal dis­tances *h*_*hkl*_ [normal distance of face (*hkl*)] [see Hammond *et al.* (2006 ▸) for further details]. From this, the crystal volume was calculated by summing all the pyramid volumes:
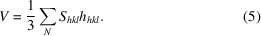
The total surface area of the crystal was obtained by summing the areas of all crystal faces:
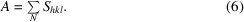
As there was only a single crystal growing in the cuvette at any one time, the solute concentration as a function of time *i* (*C_i_*) could be determined from its initial concentration (*C*_0_) and the increase in the crystal volume (*V_i_*, crystal volume at time *i*) due to absorption of the solute:
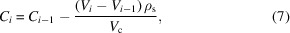
where *V*_c_ is the volume of the cuvette and ρ_s_ is the crystal density.

## 3. Results and discussion

### 3.1. Determination of the growth rates for all the 3D morphological faces

**Figure 4:**
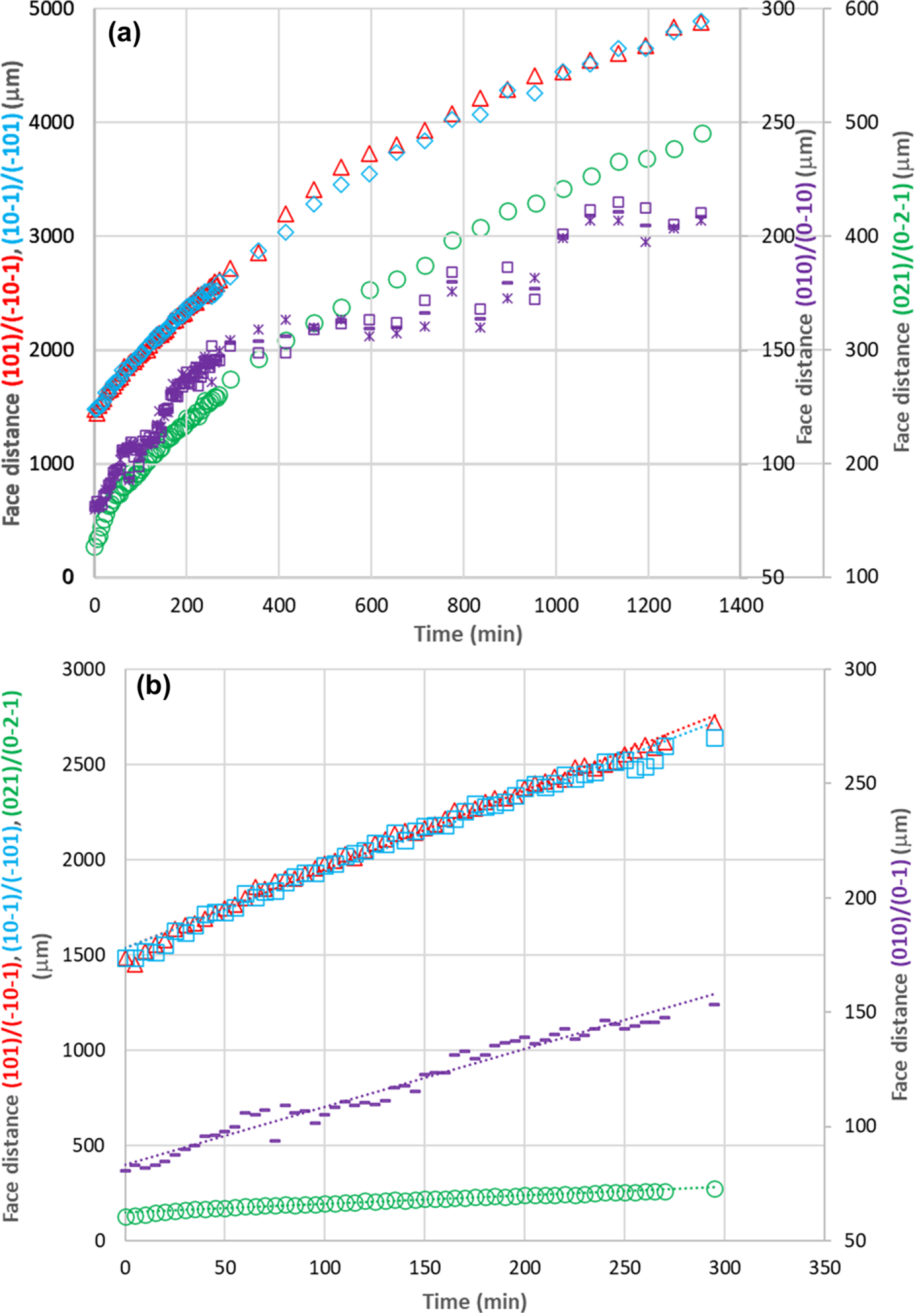
(*a*) Normal distances between paired crystal faces during crystal growth in a growth cell at σ = 1.05. The symbols represent the lengths of the (101)/(101) faces (red triangles), (101)/(101) faces (blue diamonds), (021)/(021) faces (green circles) and (010)/(010) faces [purple squares, crosses and dashes). Purple symbols show the lengths of (010)/(010) faces based on the shadow-width measurements at opposite edges: δ_1_ (squares), δ_2_ (crosses) and their average (dashes). (*b*) Initial growth-rate kinetic data highlighting the early-stage normal distances (lengths) used for growth-rate fittings at σ = 1.05. The symbols represent the lengths of (101)/(101) faces (red triangles), (101)/(101) faces (blue squares), (021)/(021) faces (green circles) and (010)/(010) faces (purple dashes), with dotted lines indicating the linear fits.

A comparison of the crystal length measurements is given in Fig. 4 ▸(*a*). The two crystallographically equivalent capping face measurements were found to be close throughout. The average relative differences of the estimated heights in the {010} face normal direction based on the two shadow widths were found to be quite small in the initial stages of the growth process (2.1% for 0–295 min) when compared with the larger value of 4.2% during the later stages (295–1315 min). From further examination of the crystal images (Fig. S2) captured with a tilting angle of 29° with respect to the vertical position of the microscopic camera, the top face (021) and bottom face (021) show a relatively small difference, resulting in the difference between *d*_(010)_ and *d*_(0
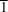
0)_, hence their normal growth rates, being also quite small. This supports, to an extent, the assumption that the two prismatic faces (021) and (021) have developed symmetrically, and also that the growth rates of top (010) and bottom (010) faces were quite symmetric. However, this may only be treated as a special case, as different surface chemistry because of fluctuations in the local growth environments surrounding the faces due to differences in the mass transfer of solute may also lead to differences in the estimates of these heights.

The crystal facet lengths as a function of time from the early stage of the growth process (0–295 min) are given in Fig. 4 ▸(*b*) along with linear fittings. The goodness of fit values (*R*^2^) are 0.99, 0.99, 0.98 and 0.97 for capping faces (101)/(101) and (101)/(101), prismatic faces (021)/(021), and basal plane faces (010)/(010), respectively.

Table 2 ▸ lists the habit-face-based crystal growth rates of capping faces (101)/(101) and (101)/(101), prismatic faces (021)/(021), and basal plane faces (010)/(010) at a σ of 1.05. For comparison, the table also provides the capping and prismatic faces (Jiang *et al.*, 2024 ▸) under reflection light mode, illustrating the broad agreement between the measurements using transmission and reflection light modes. The growth rates of the three faces (101)/(101), (101)/(101) and (021)/(021) in the current study were found to be roughly within the error bars (Jiang *et al.*, 2024 ▸), with these variations possibly being consistent with the variations of seed sizes and the effects of potential growth-rate dispersion. The aspect ratios of morphological growth rates between the capping and prismatic faces and between the capping and basal plane faces were typically found to be close to 9 and 16, respectively. This echoes the findings of Kitamura & Ishizu (1998 ▸), who showed that the prismatic faces tend to grow slowly and exhibit a significant degree of distance variation. This makes the the estimation of meaningful growth kinetics from their experiments challenging, as shown also by the recent literature (Jiang *et al.*, 2024 ▸). The growth data from in-process β-LGA crystal images in batch crystallizers (Wang *et al.*, 2007 ▸) also highlight that there appears to be more scattering associated with the growth data for the prismatic faces when compared with the capping faces. The basal plane {010} faces were found to grow slower than the prismatic {021} faces. This echoes the observations by Kitamura & Ishizu (1998 ▸, 2000 ▸), who reported that even the {021} faces grew too slowly to be reliably measured in order to be able to obtain meaningful growth-rate data. Nonetheless, in the present work, the growth rate of the {010} faces of β-LGA crystal surfaces could be estimated for the first time.

As shown in Fig. 4 ▸(*a*), the {010} faces were found to grow from 80 to 209 µm in contrast to 127 to 490 µm for the {021} faces, indicating that the height of the crystal in the {010} face direction is about half the height in the direction of the prismatic faces. Furthermore, according to previous modelling (Turner *et al.*, 2022 ▸), previous experimental studies (Davey, 1986 ▸; Calderon De Anda *et al.*, 2005*a* ▸,*b* ▸,*c* ▸; Kitamura & Ishizu, 1998 ▸; Li *et al.*, 2006 ▸, 2008 ▸; Ma & Wang, 2012 ▸; Ma *et al.*, 2007 ▸; Ochsenbein *et al.*, 2014 ▸, 2015 ▸; Wang *et al.*, 2007 ▸) and the current work, the data confirm that the normal distances of the capping {101} faces are the largest, followed by the prismatic {021} faces, with the basal {010} faces being the smallest. It can be quite difficult to directly measure the dimension in the basal {010} direction, *i.e.* without image analysis, as the crystal image was recorded only in two dimensions. The estimated growth rate of the {010} faces was found to be ∼0.21 × 10^−8^ ms^−1^ (Table 2 ▸), which is also about half that of the prismatic {021} faces (0.37 × 10^−8^ ms^−1^). As shown in Fig. 4 ▸(*a*), the shadow distances of faces (021) and (021), δ_1_ and δ_2_ (Fig. 3 ▸), were found to have consistently small differences (on average <1.5 µm or <2.6%). The estimated tilt angles using these differences were found to be 0.3° on average, supporting the assumption that the seed crystal was lying almost completely parallel to the lower surface of the cuvette and that there are no significant fluctuations in local growth conditions at opposing faces.

### 3.2. Evaluation of the morphological evolution during growth

**Figure 5:**
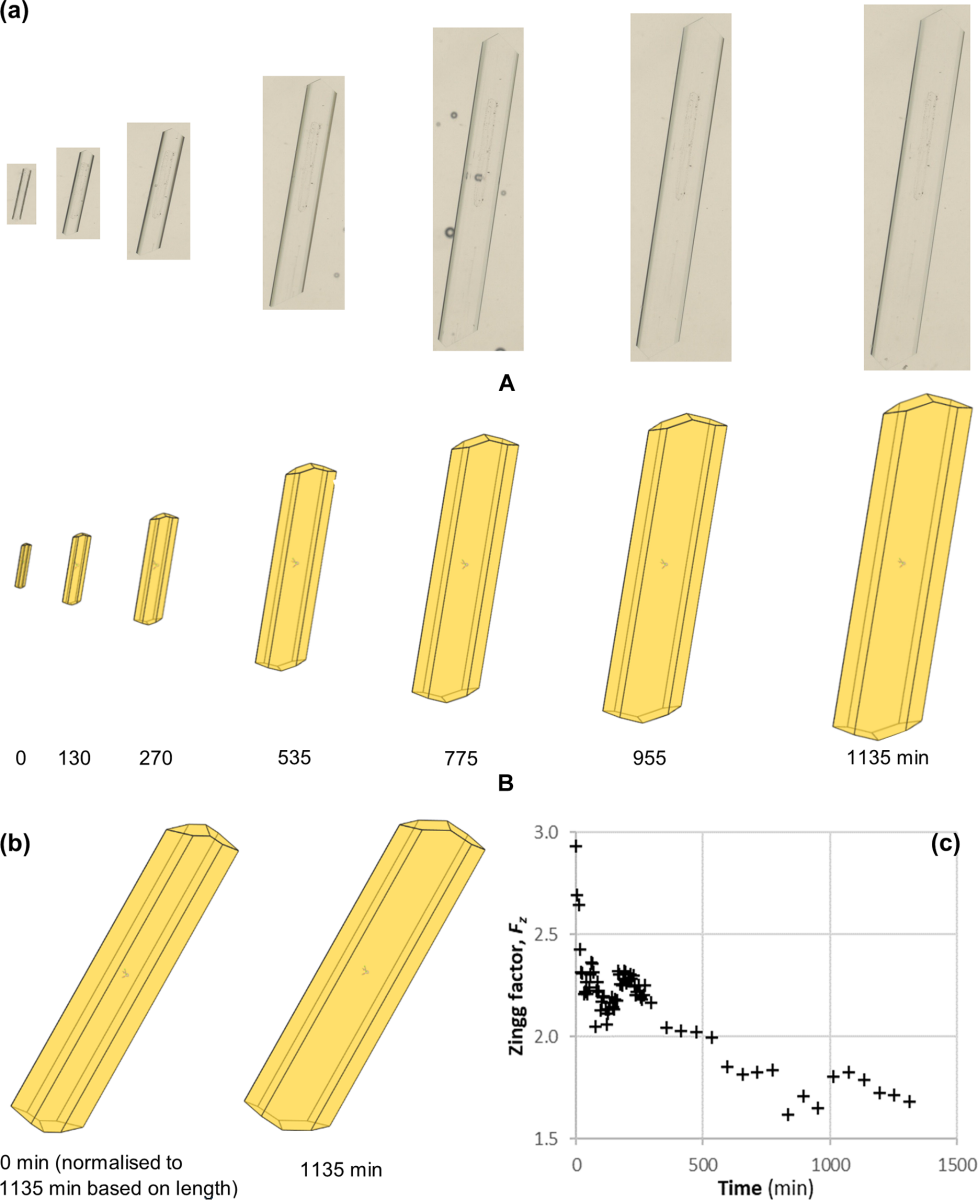
Temporal crystal growth evolution data in three dimensions at various growth times (0, 130, 270, 535, 775, 955 and 1135 min) highlighting (*a*) 2D microscopic images (A) and processing crystal size/shape (B), (*b*) shape, and (*c*) Zingg factor as a function of growth time. Enlarged versions of the 2D microscopic images in (*a*)(A) are shown in Fig. S3 to clearly visualize all of the crystal edges at the seven growth times.

The typical crystal size and shape evolution for the β-LGA crystals at different growth times (0, 130, 270, 535, 775, 955 and 1135 min) together with the associated time-dependent Zingg factor are shown in Fig. 5 ▸. The increase of the relative surface area of the capping {101} faces demonstrated the 3D crystal shape evolution [Fig. 5 ▸(*a*)B] against time when compared with the 2D crystal images [Fig. 5 ▸(*a*)A]. Reflecting the different growth kinetics of the different morphological forms, the crystals at this relative solution supersaturation were found to become more tabular with time due to the slower growth of the basal plane {010} surfaces with respect to the other habit faces. This change in shape is highlighted by the calculated Zingg factor Fz (Liu *et al.*, 2008 ▸), which was found to decrease from 2.9 to 1.7 (>1) over the growth period [Fig. 5 ▸(*c*)]. The decrease of the Zingg factor indicates needle- to tabular-like crystal shape evolution, as shown in Figs. 5 ▸(*a*) and 5 ▸(*b*). The fast decrease from 2.9 to 2.3 of Fz values for the initial five time intervals, as shown in Table S1 of the supporting information, was caused by the fast growth of face {021}. This may indicate the possible existence of some degree of inhomogeneity in surface properties on these three faces, in particular the face {021}, through surface imperfection and/or surface contamination of the seed crystal.

### 3.3. Assessment of solution de-supersaturation due to crystal growth

**Figure 6:**
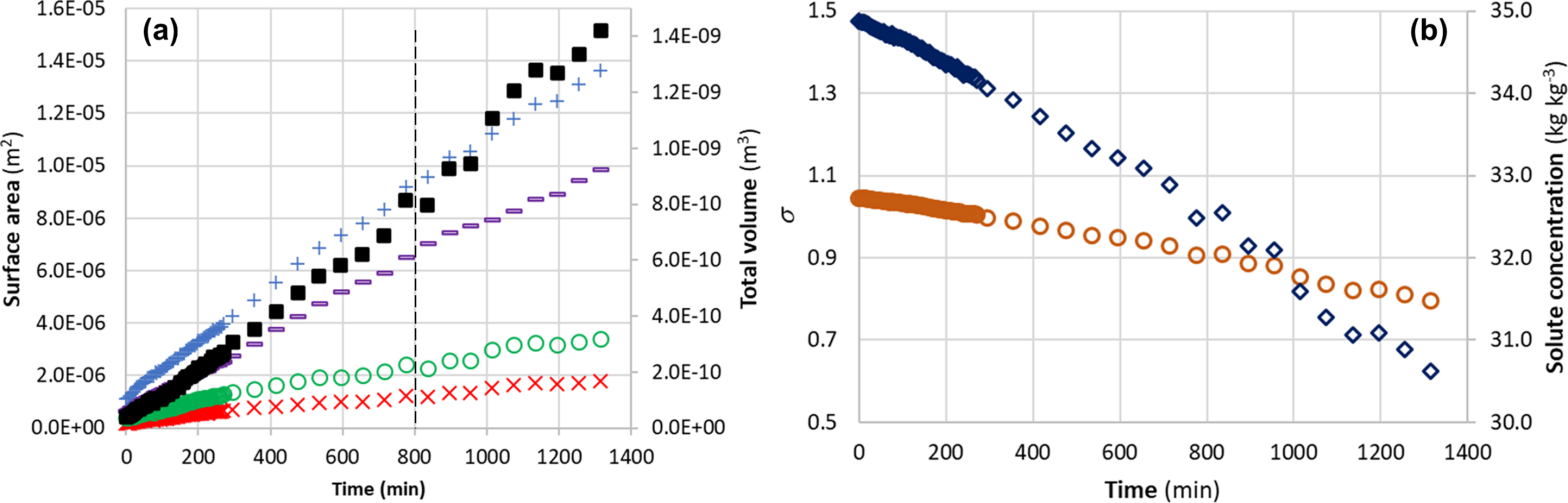
(*a*) Surface areas of faces {101} (×5) (crosses), {021} (circles) and {010} (dashes), and total surface area (plus symbols) and volume (filled squares) of the evolving β-LGA crystal. (*b*) Variations of solute concentration (diamonds) and relative supersaturation (circles) during crystal growth in the growth cell.

Fig. 6 ▸(*a*) shows the surface-area variations for the {101}, {021} and {010} faces during the growth period, together with the whole crystal surface area and volume. The associated estimated solute concentration and relative time-dependent solution supersaturation calculated from the equilibrium solubility and calculated crystal volume are given in Fig. 6 ▸(*b*). All the facet surface areas and crystal volumes were found to increase almost linearly with time (*R*^2^ > 0.98 for all linear fits), though the crystal volume was found to increase slightly faster after ∼800 min [vertical dashed line in Fig. 6 ▸(*a*)], which may reflect the fact that the data were more scattered from the manual image processing and that there was a larger time step (60 min) between data points compared with the early stages (5 min) of crystal growth. During the crystal growth process, the solute concentration was found to correspondingly decrease linearly with time, leading to a fairly linear decrease of σ from the initial value of 1.05 down to ∼0.8. However, during the early stages (0–295 min), σ was still quite close to the initial value of 1.05, hence supporting the initial-rate assumption of the method (Jiang *et al.*, 2024 ▸) for determining the growth rate. This approach is helpful in that it highlights the opportunity to potentially fit the growth-rate kinetic data over a wider range of supersaturation.

## 4. Conclusions and future work

The full 3D facetted growth of β-LGA has been monitored and characterized *in situ* with a temperature-controlled crystal growth cell using transmission optical microscopy. The measured growth rates of the capping {101} and prismatic {021} faces were found to be consistent with those determined using reflection light mode (Jiang *et al.*, 2024 ▸), *i.e.* within the standard deviations of five repeated experiments at σ = 1.05 with the reflection mode. The growth rate in the basal plane {010} faces was also measured for the first time and found to be about half that of the prismatic {021} faces. Overall, the LGA morphology was found to evolve with time from a needle-like morphology in the early stages of growth to a more tabular crystal habit, reflecting the fact that crystal growth would appear to be constrained by the slower-growing basal {010} plane, as highlighted by a corresponding decrease in Zingg factor with time. The increase in surface area of faces {101}, {021} and {010} during the growth cycle and that of the corresponding crystal volume leads to a decrease in solute concentration and hence solution supersaturation. The slow decrease of supersaturation at the early stages of the growth process supports the initial-rate approach used for kinetics assessment.

Further work is underway to develop an automated process to analyse, *in situ*, the facet growth rates of all crystal habit faces in three dimensions, encompassing various orientations and lighting conditions. We intend to use a 3D rendering engine (*Blender*; Blender Online Community, 2022 ▸) and a synthetic dataset of 3D crystal shapes created using crystallographic and molecular modelling software, *e.g.HABIT98* (Clydesdale *et al.*, 1991 ▸, 1996 ▸) and *SHAPE* (Dowty, 2023 ▸). The detailed methodology and corresponding results will be published in due course. This fully automated 3D approach will enable monitoring in real time of the 3D dynamics of the overall crystallization process, for which the current work will provide a key case study. Overall, this research forms part of wider efforts into the development of digital crystallization process engineering, encompassing its integrated MPB modelling (Ma & Roberts, 2018 ▸, 2019 ▸; Ma *et al.*, 2008 ▸) and computational fluid dynamics studies of industrial-scale crystallizer hydro­dynamics (Camacho Corzo *et al.*, 2020 ▸).

## Supplementary Material

Supporting information. DOI: 10.1107/S1600576724008173/ui5011sup1.pdf

## Figures and Tables

**Table 1:** Characteristic crystallographic structural data for β-LGA, important for the prediction of crystal morphology

Material descriptor	β-LGA (Lehmann *et al.*, 1972 ▸)
Refcode	LGLUAC11
Space group	*P*2_1_2_1_2_1_
*Z*/*Z*′	4/1
*a* (Å)	5.159 (5)
*b* (Å)	17.300 (2)
*c* (Å)	6.948 (7)
α (°)	90
β (°)	90
γ (°)	90
Unit-cell volume (Å^3^)	620.114

**Table 2:** Facet growth rates of all three faces at a σ of 1.05, with the results from reflection light mode (Jiang *et al.*, 2024 ▸) for comparison

Growth temperature (°C)	Relative supersaturation σ	Growth rate (×10^−8^ ms^−1^)				Lighting mode	Reference
		(021)/(021)	(101)/(101)	(101)/(101)	(010)/(010)		
46	1.05	0.37	3.45	3.34	0.21	Transmission	This study
46	1.05	0.37	3.71	3.89	N/A	Reflection	Jiang *et al.* (2024 ▸)
