# Supplementary material for: Deconstructing 3D growth rates from transmission microscopy images of facetted crystals as captured in situ within supersaturated aqueous solutions
Source: J Appl Crystallogr. 2024 Sep 25;57(Pt 5):1557–65. doi: 10.1107/S1600576724008173 (PMC11460390; doi:10.1107/S1600576724008173)
Supplement: Supplementary file 1 [file j-57-01557-sup1.pdf]

JOURNAL OF
APPLIED
CRYSTALLOGRAPHY

Volume 57 (2024)

Supporting information for article:

Deconstructing 3D growth rates from transmission microscopy images of facetted crystals captured *in situ* within supersaturated aqueous solutions

Cai Y. Ma, Chen Jiang, Thomas P. Ilett, Thomas A. Hazlehurst, David C. Hogg and Kevin J. Roberts

The supplementary material supports the main manuscript by providing further details of the following: **Figure S1** presents the crystal images captured using Keyence VHX7000 digital microscope in a transmission light mode without and with a diffuser. **Figure S2** presents the contributions of the different molecular fragments to the lattice energies of the two forms. Figure S3 shows the selected crystal images at growth times of 0, 130, 270, 535, 775, 955 and 1135 min being enlarged 420 times to clearly visuallise all of the crystal edges. **Table S1** presents the lengths, widths and heights of the first 5 time intervals used for the calculation of *Fz*.

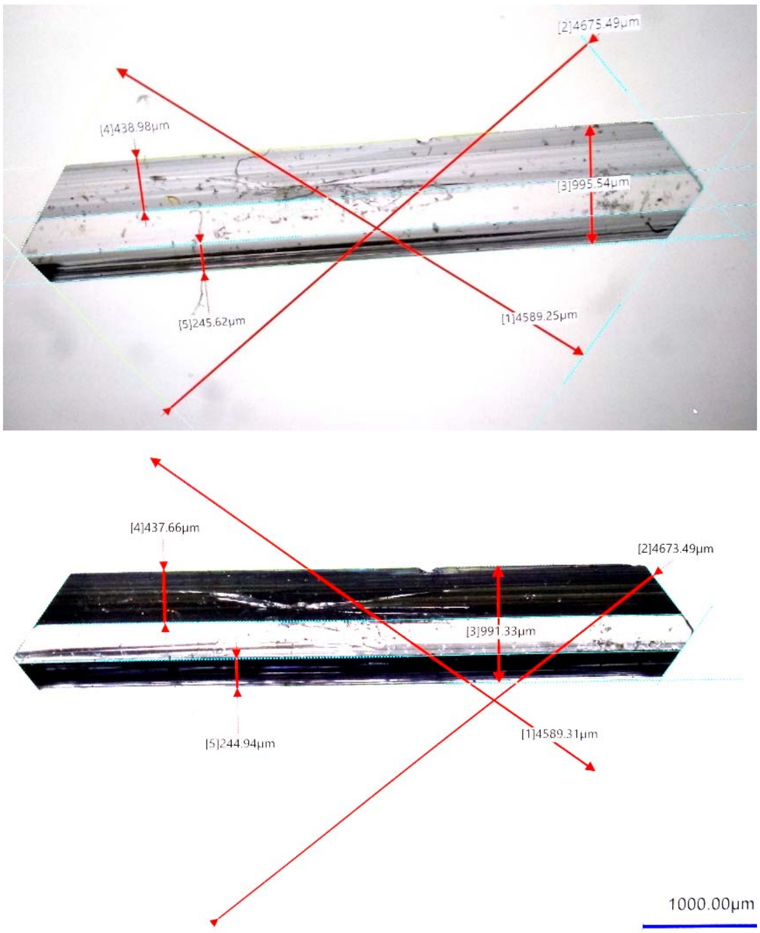

Figure S1 Crystal images captured using Keyence VHX7000 digital microscope in a transmission light mode without and with a diffuser. The measured distances were found to be similar.

Based on the crystal images (**Figure S2**) captured with a tilting angle of 29° with respect to vertical position of the microscopic camera, the top face (021) and bottom face (0-21) show a relatively small difference, resulting in the difference between $d_{(010)}$ and $d_{(0\text{–}10)}$, hence their normal growth rates, being also quit small. As only the crystal images from the early stage of the growth data (in this study up to 180 min) were used for the calculation of facet growth rates (Jiang *et al.*, 2024), the thin intermediate layer of solution may provide the required solute molecules for the early stage growth of face (0-10). The Keyence VHX7000 digital microscope was operated in transmission light mode with zero tilting angle to provide vertical incidence of LED lighting. The light beam has a diameter of about 20 mm which is > 20 times bigger than the growing crystal located at the centre of the beam, hence the light beam tending to be parallel though some divergence may still exist under a diffuser. Test runs have been carried out and, as shown in **Figure S2**, the captured crystal images confirmed the limited effect of possible light divergence on the crystal edges recorded.

The five distances (**Figure 3(a)**) measured with and without a diffuser were found to be very similar (< 1% difference), hence indicating the vertical light being quite parallel for capturing the crystal images.

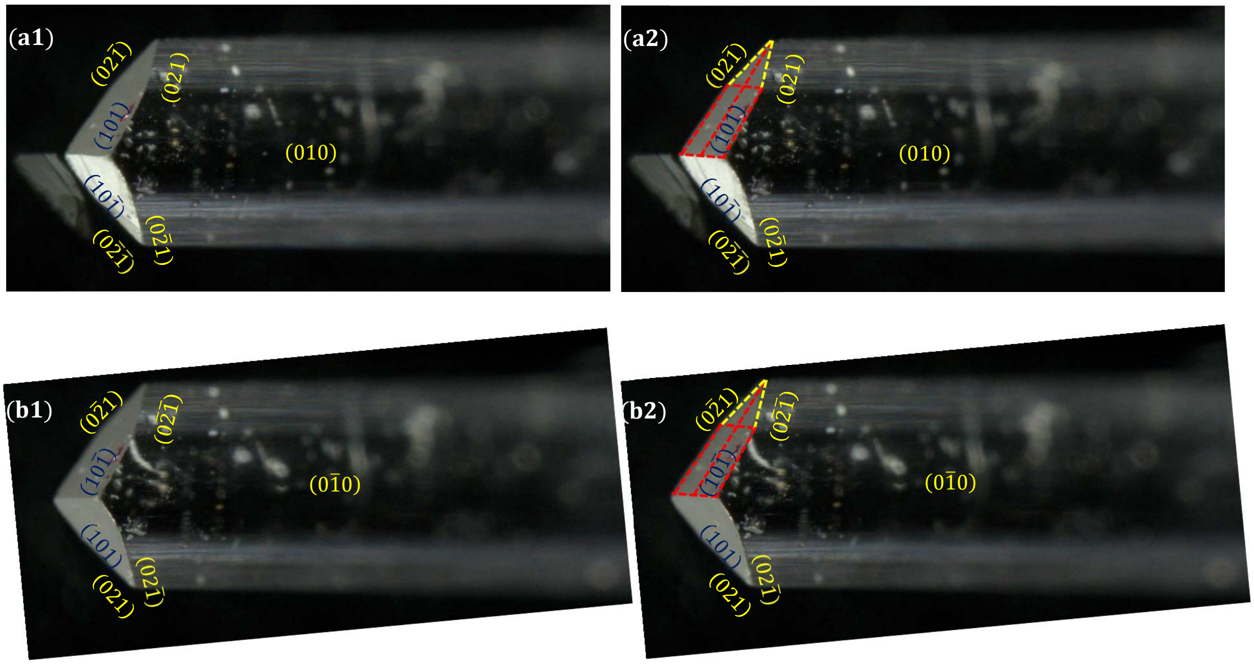

Figure S2 Crystal images captured by the Keyence VHX7000 digital microscope with a tilting angle of 29°: (a1, a2) image of a β-form LGA crystal under an orientation of face (010) on top view

with the dashed lines in (a2) for eye guide; (b1, b2) image of the same crystal under an orientation of face (0-10) on top view with the dashed lines in (b2) for eye guide.

The selected crystals images (**Fig. 3(a)(A)**) captured during the crystal growth process in this study are enlarged, as shown in **Fig. S3,** to visualise all of the crystal edges.

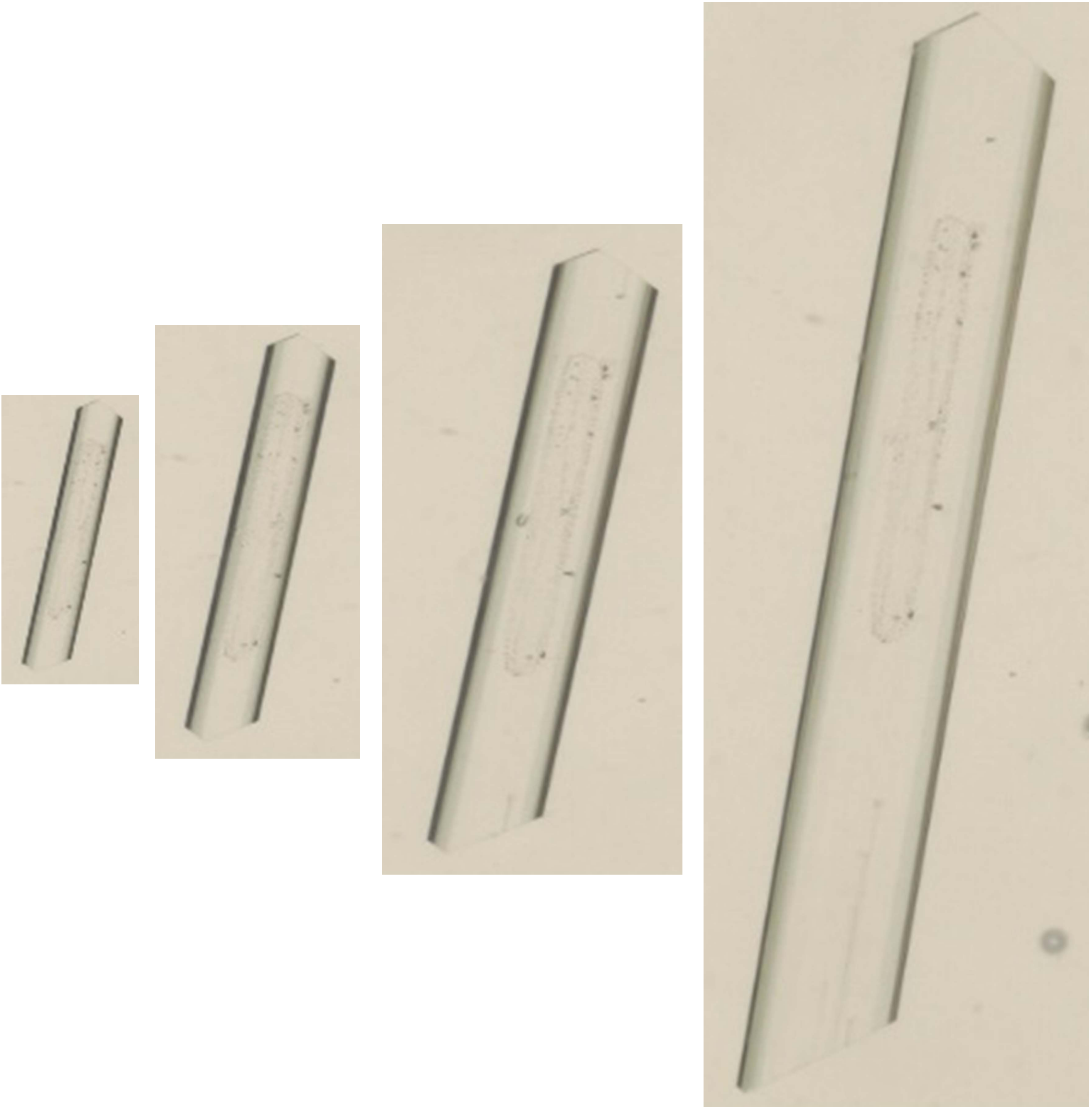

0 min **130 min** **270 min** **535 min**

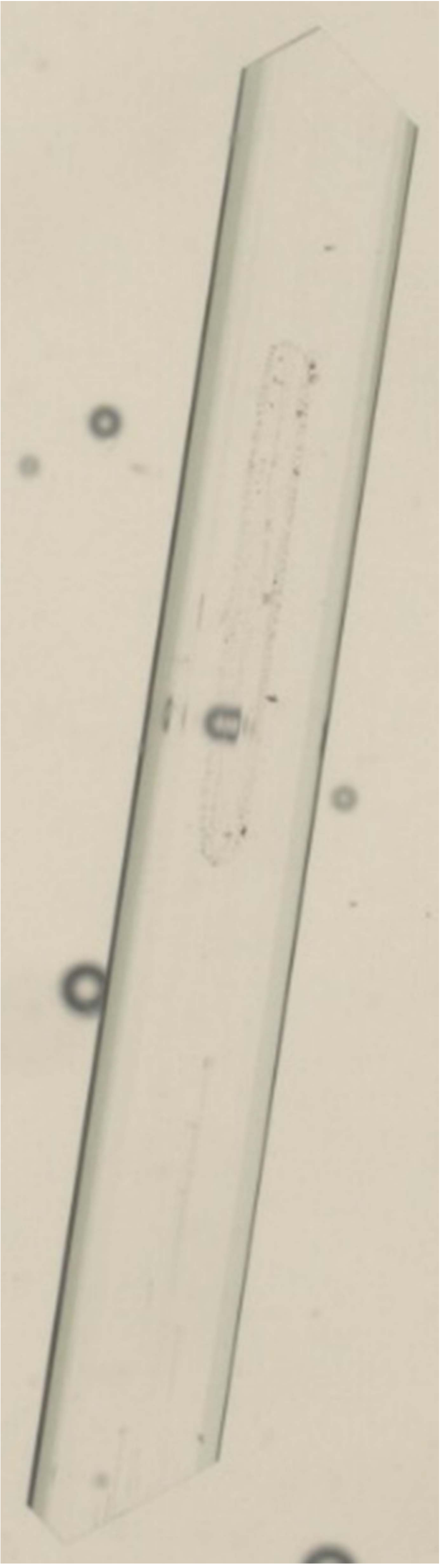

775 min

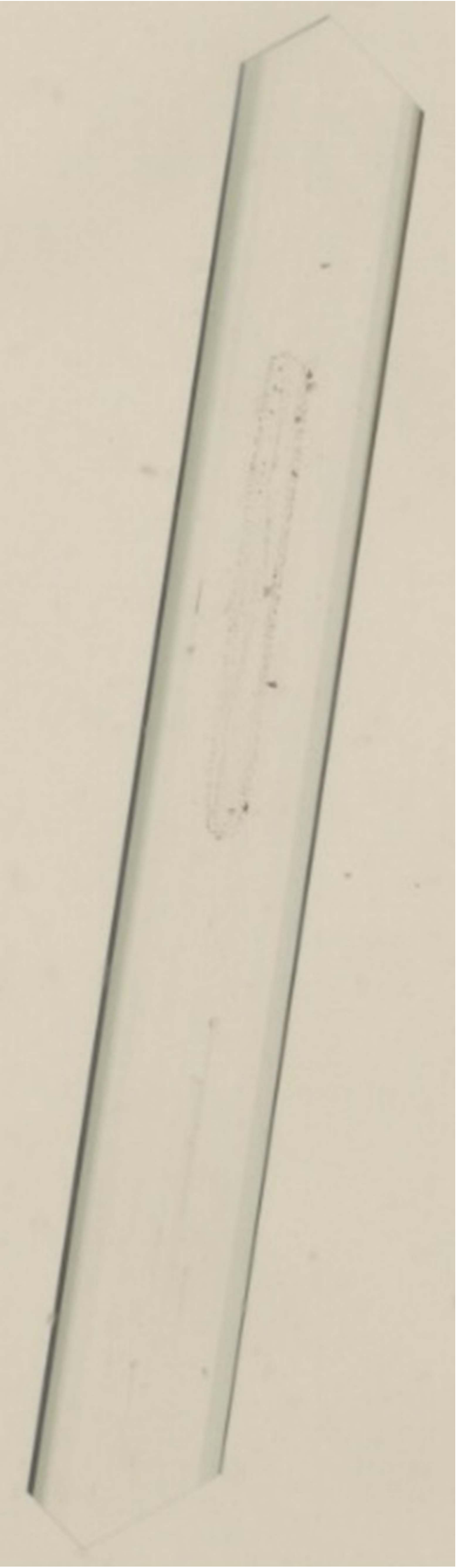

955 min

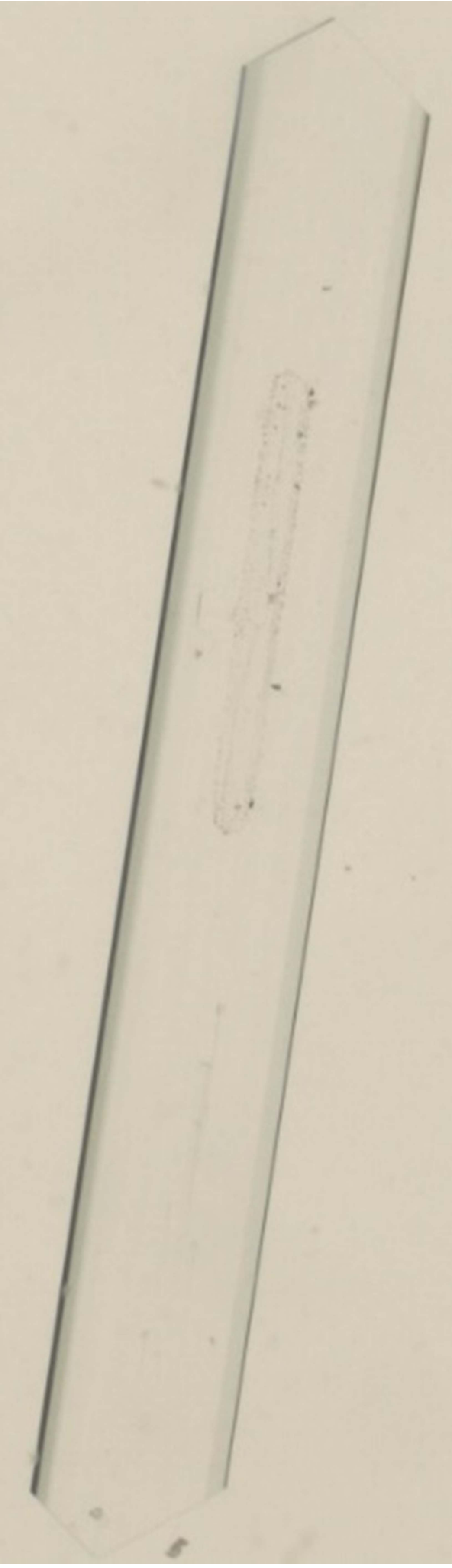

1135 min

Figure S3 The selected crystal images (Fig. (3)(A)) were enlarged by 420 times to clearly visualise all of the crystal edges at growth times of 0, 130, 270, 535, 775, 955 and 1135 min.

Examining the results from the first 5 time intervals (**Table S1**) indicates the width (related to face {021}) growing 1.18 times, the length (related to face {101}) 1.06 times and the height (related to face {010}) 1.05 times between 0 to 20 min, hence the *Fz* values dropping rapidly from 2.9 to 2.3. This might indicate the possible existence of some degrees of inhomogeneous surface properties on these three faces, in particular the face {021}, through surface imperfection and/or surface contamination of the seed crystal. Further research is needed to explore and potentially confirm this.

Table S1 The lengths, widths and heights of the first 5 time intervals used for the calculation of *Fz*.

Time (min)	L (mm)	H (mm)	W (mm)	*Fz*
0	1483	81	202	2.9
5	1468	83	213	2.7
10	1520	82	217	2.6
15	1534	83	229	2.4
20	1566	85	240	2.3

Jiang, C., Ma, C. Y., Hazlehurst, T. A., Ilett, T. P., Jackson, A. S. M., Hogg, D. C. & Roberts, K. J. (2024). *Crystal Growth & Design* **24**, 3277– 3288.
